# Maintenance of Increased Mouth Opening in Oral Submucous Fibrosis Patient Treated with Nasolabial Flap Technique

**DOI:** 10.1155/2014/842578

**Published:** 2014-03-05

**Authors:** Milind Naphade, Bhushan Bhagat, Dwarkadas Adwani, Ranjit Mandwe

**Affiliations:** Department of Oral and Maxillofacial Surgery, V.Y.W.S. Dental College and Hospital, Wadali Road, Amravati 444602, India

## Abstract

Oral submucous fibrosis (OSMF) is an insidious chronic disease affecting any part of the oral cavity and sometimes the pharynx with epithelial atrophy leading to stiffness of the oral mucosa, causing trismus and inability to eat. However, a more serious complication of this disease is the risk of the development of oral carcinoma. A case of OSMF reported with initial interincisal mouth opening; 8 mm which was treated surgically with nasolabial flap technique followed by active mouth opening exercises for 6 months with Hister's jaw exerciser. The patient could maintain mouth opening of 32 mm at the end of 18-months followup. The patient was observed closely for any malignant changes in the oral cavity.

## 1. Introduction

Oral submucous fibrosis (OSMF) is an insidious chronic disease affecting any part of the oral cavity and sometimes the pharynx. Although occasionally preceded by and or associated with vesicle formation, it is always associated with juxtaepithelial inflammatory reaction followed by a fibroelastic change of the lamina propria, with epithelial atrophy leading to stiffness of the oral mucosa, causing trismus and inability to eat [1]. OSMF has a high rate of morbidity because it causes progressive inability to open the mouth, resulting in inability to eat and consequent nutritional deficiencies [2]. Mortality rate is significant because it transforms into oral cancer, particularly squamous cell carcinoma at a rate of 7%–30% [2].

Management includes cessation of habit and surgical release of fibrous bands followed by forceful opening of the mouth by coronoidectomy and coverage of surgical defects with nasolabial flap and postoperative active jaw physiotherapy for 6 months [3]. Surgery may induce scar tissue which reduces mouth opening due to scar contraction in mouth closing muscles [4]. Relapse is a common complication that occurs after surgical release of the oral trismus caused by OSMF [5]. A variety of jaw opening devices have been used to treat trismus [6].

The purpose of this paper is to report a definite treatment approach that combines surgery with active physiotherapy to improve the jaw opening and to prevent relapse. Small effort has been made in the present study aiming to endure adequate, functional disease free mouth opening and to detect any developing malignant change at its earliest.

## 2. Case Report

**Figure 1:**
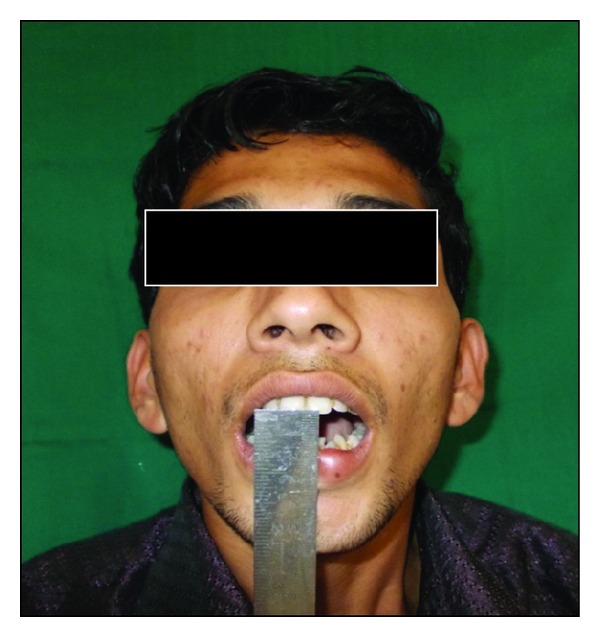
Preoperative mouth opening.

A 24-year-old male patient from India reported with a complaint of increasing difficulty of mouth opening and mastication for the previous 3 years. The patient had a habit of chewing betel nuts four times a day for 5 to 6 years. He would keep the betel nut in the mouth, against the cheeks for approximately 30 minutes each time, chew, and finally spit it out. The patient stopped this habit completely 2 years before. After histopathological confirmation of the diagnosis of oral submucous fibrosis, an informed consent was procured for the procedure of bilateral fiberotomy followed by coronoidectomy and closure of the intraoral surgical defect by nasolabial flap. Routine preanesthetic investigations were done. Initial interincisal mouth opening recorded was 8 mm (Figure 1).

**Figure 2:**
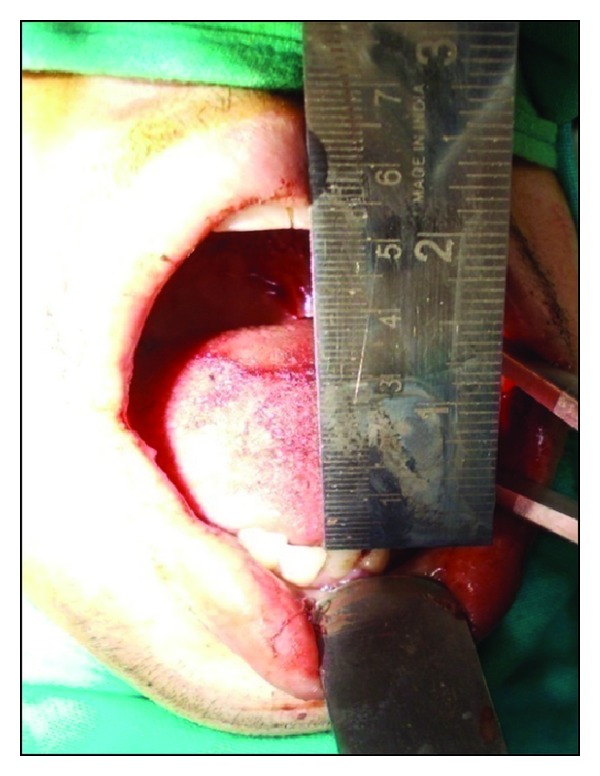
Intraoperative mouth opening.

**Figure 3:**
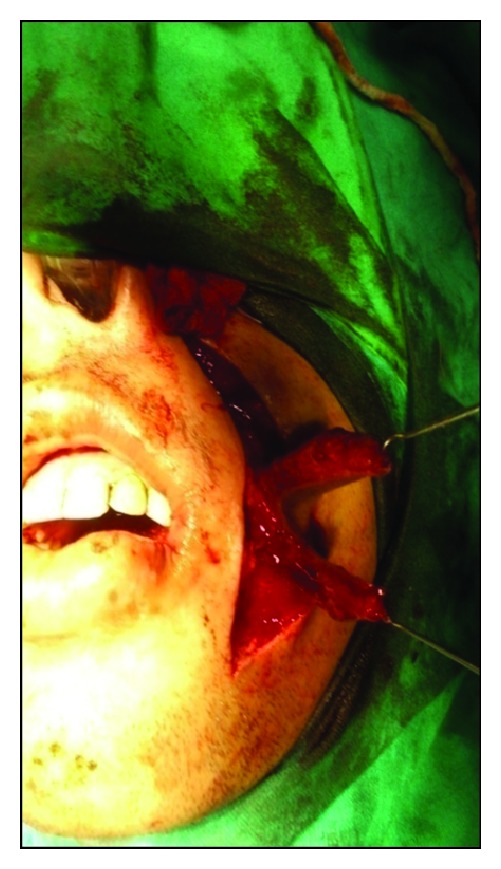
Harvesting of nasolabial flap.

**Figure 4:**
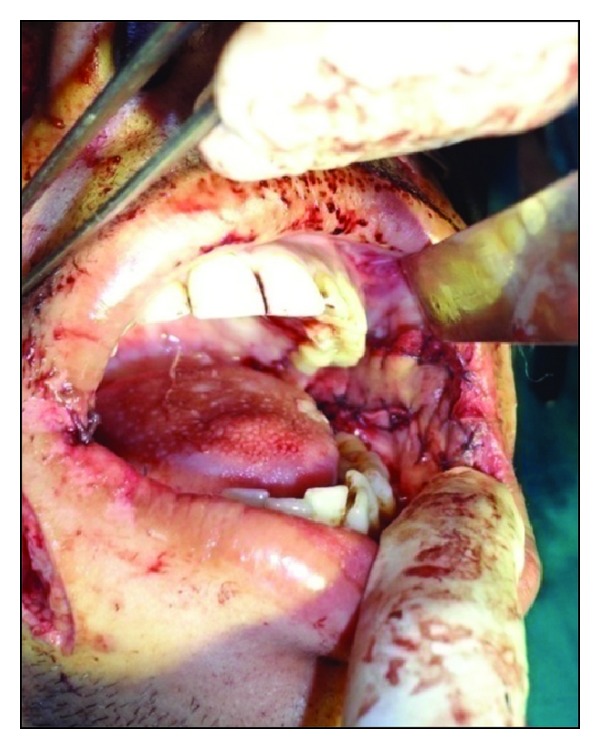
Flap sutured.

**Figure 5:**
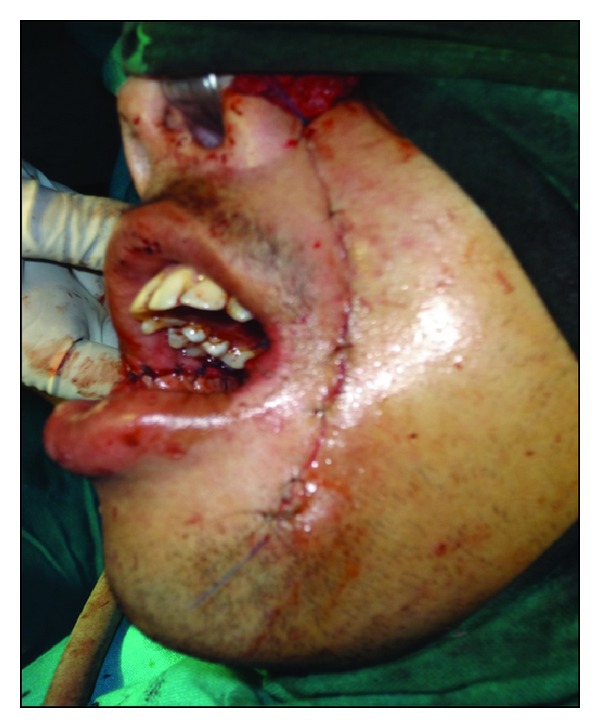
Extra oral suturing.

Under aseptic precautions, awake fibreoptic nasotracheal intubation was used for administration of general anesthesia. Incisions were made by using an electrosurgical knife from the corner of mouth to the soft palate at a level of the linea alba, avoiding injury to the duct of parotid gland. Fiberotomy of the bands was done. The coronoid processes were approached through the same incision and a bilateral coronoidectomy was carried out. The maxillary and mandibular third molars were extracted. Intraoperative interincisal distance was recorded (Figure 2). Nasolabial flaps were raised (Figure 3) bilaterally in the plane of superficial muscular aponeurotic system from both terminal points to the region of the central pedicle. The pedicle was 1 cm lateral to the corner of mouth and the diameter of the pedicle was roughly 1 cm. The flap was transposed intraorally through a small transbuccal tunnel near the commissure of mouth with no tension. The inferior wing of the flap was sutured to the anterior edge of the defect, while the superior wing was sutured to the posterior edge of defect (Figure 4). The extraoral defect was closed primarily in layers after liberal undermining of the skin in the subcutaneous plane to prevent any tension across the suture line (Figure 5). A soft temporomandibular joint trainer was placed in the oral cavity for 7 days postoperatively to prevent occlusal trauma induced dehiscence of the flap. Postoperatively, the patient received prophylactic antibiotics and nasogastric feeding for 1 week.

**Figure 6:**
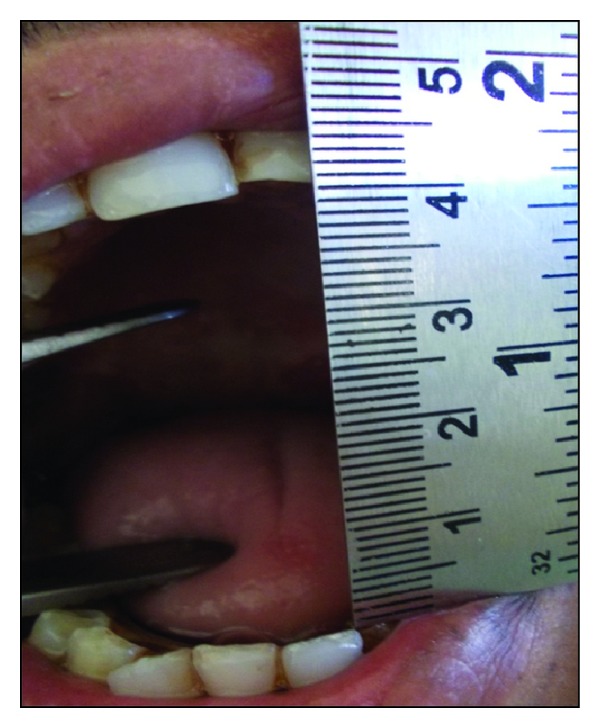
Postoperative mouth opening after 18 months.

The oral physiotherapy was started after 48 hours with the help of wooden spatulas for 1 week followed by a Hister's jaw exerciser to prevent contracture and relapse. The patient was instructed and motivated to do physiotherapy himself for up to 6 months. The mouth opening could be maintained to 32 mm by the end of 18 months (Figure 6) and was followed closely to notice any malignant changes in oral cavity.

## 3. Discussion

OSMF is multifactorial in origin affecting 5 million people in India alone (0.5% of the Indian population) [2]. Prevalence of OSMF in Maharashtra (India) is 21.9/1000. Male predominates with the ratio of 4.9 : 1 [7]. OSMF is poorly understood and unsatisfactorily treated disease. However, a more serious complication of this disease is the risk of the development of oral carcinoma. The precancerous nature of OSMF has been observed with development of slowly growing squamous cell carcinoma in one-third of OSMF patients [2, 7].

Various treatment modalities like medical and/or surgical tried to improve the patient condition [2, 8]. Medical treatment is palliative therapy which is not going to reverse the condition completely. Surgical treatment modality has its own advantage and disadvantage.

Nasolabial technique flap has versatility and a distinct edge in maintaining the mouth opening in long run and is advent over the other being local, easily, accessible and modifiable with single sitting operation. Linear closure of donor site is possible resulting in well-camouflaged scar in the nasolabial fold [3]. Large defects can be closed. Minor complications include increase in intercommisure width and the intraoral hair growth [3]. Exercises are frequently proposed to prevent or treat trismus, including active range of motion exercises combined with passive range of motion exercises [5].

The case illustrates the relentless progression of OSMF and its significant morbidity and mortality, and it also emphasizes the importance of close followup of such cases. Because of significant cancer risk among these patients, periodic biopsies of suspicious regions of oral mucosa are essential for early detection and management of high risk oral premalignant lesions and prevention of cancer [9].

## 4. Conclusion

The single most important factor that is known to improve the end result of head and neck cancer is the early diagnosis of these cancers while they are still localized and can be treated with a high probability of cure. Early diagnosis of cancer plays a lifesaving pivotal role in overall management.

Management of OSMF by nasolabial flap technique with postoperative exercises enables gaining functional disease-free mouth opening with minimal functional and cosmetic deformity at the donor site. This can be very useful aid for “early diagnosis” of cancer. This is perhaps the most important service we can provide.

## References

[1] Pillai R, Balaram P, Reddiar KS (1992). Pathogenesis of oral submucous fibrosis: relationship to risk factors associated with oral cancer. *Cancer*.

[2] Shevale VV, Kalra RD (2012). Management of oral sub-mucous fibrosis: a review. *Indian Journal of Dental Sciences*.

[3] Borle RM, Nimonkar PV, Rajan R (2009). Extended nasolabial flaps in the management of oral submucous fibrosis. *British Journal of Oral and Maxillofacial Surgery*.

[4] Dijkstra PU, Sterken MW, Pater R, Spijkervet FKL, Roodenburg JLN (2007). Exercise therapy for trismus in head and neck cancer. *Oral Oncology*.

[5] Le PV, Gornitsky M (1996). Oral stent as treatment adjunct for oral submucous fibrosis. *Oral Surgery, Oral Medicine, Oral Pathology, Oral Radiology, and Endodontics*.

[6] Stubblefield MD, Manfield L, Riedel ER (2010). A preliminary report on the efficacy of a dynamic jaw opening device (dynasplint trismus system) as part of the multimodal treatment of trismus in patients with head and neck cancer. *Archives of Physical Medicine and Rehabilitation*.

[7] Hazarey VK, Erlewad DM, Mundhe KA, Ughade SN (2007). Oral submucous fibrosis: study of 1000 cases from central India. *Journal of Oral Pathology and Medicine*.

[8] Adwani DG (1982). *Histopathological studies before and after Kenacort in oral submucous fibrosis [MDS thesis]*.

[9] Naphade MV, Naphade UM (2011). Major immunoglobulin status and lactate dehydrogenase isozyme profile in oral premalignancy and malignancy. *Dental Dialogue Official Journal of IDA, MSB*.

